# Engineering Students as Co-creators in an Ethics of Technology Course

**DOI:** 10.1007/s11948-021-00326-5

**Published:** 2021-07-23

**Authors:** Gunter Bombaerts, Karolina Doulougeri, Shelly Tsui, Erik Laes, Andreas Spahn, Diana Adela Martin

**Affiliations:** 1 Philosophy and Ethics, Department IE&IS, Eindhoven University of Technology, Eindhoven, The Netherlands; 2 Eindhoven School of Education (ESoE), Eindhoven University of Technology, Eindhoven, The Netherlands; 3 VITO – Vision on Technology, Boeretang 200, 2400 Mol, Belgium; 4 College of Engineering and Built Environment, Technological University Dublin, Dublin, Ireland

**Keywords:** Engineering-ethics education, Effectiveness model, Challenge-based learning, Co-creation, Self-determination theory, Competence development

## Abstract

Research on the effectiveness of case studies in teaching engineering ethics in higher education is underdeveloped. To add to our knowledge, we have systematically compared the outcomes of two case approaches to an undergraduate course on the ethics of technology: a detached approach using real-life cases and a challenge-based learning (CBL) approach with students and stakeholders acting as co-creators (CC). We first developed a practical typology of case-study approaches and subsequently tested an evaluation method to assess the students’ learning experiences (basic needs and motivation) and outcomes (competence development) and staff interpretations and operationalizations, seeking to answer three questions: (1) Do students in the CBL approach report higher basic needs, motivation and competence development compared to their peers in the detached approach? (2) What is the relationship between student-perceived co-creation and their basic needs, motivation and competence development? And (3) what are the implications of CBL/CC for engineering-ethics teaching and learning? Our mixed methods analysis favored CBL as it best supported teaching and research goals while satisfying the students’ basic needs and promoting intrinsic motivation and communication competences. Competence progress in other areas did not differ between approaches, and motivation in terms of identified regulation was lower for CBL, with staff perceiving a higher workload. We propose that our case typology model is useful and that as a method to engage students as co-creators, CBL certainly merits further development and evaluation, as does our effectiveness analysis for engineering ethics instruction in general and for case-study approaches in particular.

## Introduction

Case studies are popular in engineering-ethics education and the variation in approaches is considerable (Colby & Sullivan, 2008; Haws, 2001; Herkert, 2000). Several studies analyzing such approaches in higher education addressed their effects on student motivation (Bairaktarova & Woodcock, 2017; Colby & Sullivan, 2008; Fotheringham, 2008; Haws, 2001; Herkert, 2000; Wilson, 2013). Yet, despite the widespread use of case instruction and these first inquiries into its impact on motivation, there is a lack of rigorous research on its effectiveness (Barry & Ohland, 2009; Bombaerts et al., 2018; Thiel et al., 2013; van Diggelen et al., 2019), leaving it unclear which approach is the more effective for which particular goal (). Accordingly, there is an imperative need to understand the principles governing the implementation of ethics case studies in engineering curricula and of developing metrics for measuring the effectiveness of various case formats and applications (Martin et al., 2021, p.13).

The present study aims to fill this gap by providing a practical typology of case-study approaches in higher engineering-ethics education and presenting the results of a mixed methods evaluation of students’ learning experiences and outcomes, and staff interpretations and operationalizations for two different approaches to an ethics-of-technology course for first-year engineering students in the Netherlands. Besides describing our findings and conclusions for the course evaluated, we will discuss potential implications for case approaches in engineering-ethics instruction in general.

## Classifying Case Approaches in Engineering-Ethics Education

### Case Studies

Described as promising scenarios for pedagogical purposes (Lundeberg, 2008), case studies have the “ability to introduce challenging, real-world situations and related decision complexity into the classroom” (Kauffmann et al., 2005), thus reflecting the features of a true profession or authentic problems professionals might encounter in everyday practice (Herreid, 1994). They have a significant contextual component, are ambiguous and allow for multiple perspectives and representations of a problem (Martin et al., 2018, 2019).

Case studies may differ substantially as to their *scope*. Although Colby and Sullivan (2008, p. 331) note that cases “typically involve a mix of normal human error, organizational failure and individual violations of professional standards”, we can distinguish between *micro* and *macro* cases, the first emphasizing the individualist perspective of an agent required to make a decision in light of the situation described in the scenario, and the latter the broader context and the collective nature of decision-making in engineering (Herkert, 2005; Martin et al., 2019). Considering the likelihood of occurrence of the scenario described, case studies may focus on *special or one-off events*, i.e. notable failures and disasters, or on more mundane, *common situations* that are more likely to occur in an engineer’s career.

We propose to distinguish an additional dimension based on the degree of student involvement, where the content of case studies can be denoted as *detached* when the scenario is remote and students have no direct experience or involvement with the case, requiring a *co-creative,* active engagement of students in manipulating the case to arrive at a specific outcome. We consider co-creative special-event cases less relevant and will discount these. Since our typology is meant to serve as a practical tool and not as a systematic delineation of case types, below we will define six case approaches (see Table 1).

**Table 1:** Practical typology of engineering-ethics cases based on three dimensions

Student Involvement	Likelihood of occurrence	Scope	Focus/Educational objectives	Examples
Detached	Special event	Macro	Responsible engineering practice	Chernobyl (Wilson, 2013: 636); Hurricane Katrina (Newberry, 2010) (Newberry, 2010); Rana Plaza collapse (Kumar, 2015), Fukushima Daiichi (Guntzburger & Pauchant, 2014); Deepwater Horizon oil rig explosion (Beever & Hess, 2016); Challenger (Vaughan, 1996); China Airlines CI-611 Accident (Tai, 2013); Ford-Pinto gas tank ignition (Velasquez, 1992)
Detached	Special event	Micro	Accountability and prevention	
Detached	Common scenario	Macro	Forward looking reflection	Millennium Goals: Developing a biodegradable fabric (Gorman et al., 2000); Nanosilver lining (Dempsey et al., 2017); water supply and demand in Dublin area (Byrne, 2012); the adoption of the fluorescent lamp (Bijker, 1997)
Detached	Common scenario	Micro	Moral reasoning and professional codes	Professional integrity, conflict of interest, safety (Abraham & Abulencia, 2011; Shallcross, 2013; this article)
Co-creative	Common scenario	Macro	Active stance in ethical aspects of technological innovation and policy	Where there is no engineer (McCarton & O'Hógáin, 2018)—Contribute to ocean plastic clean-up; global client-oriented projects (Bissett-Johnson & Radcliffe, 2021)
Co-creative	Common scenario	Micro	Active stance in ethical aspects of engineering design	Challenge-based learning (Membrillo-Hernández et al., 2019a, b; this article)

*Detached special-event macro cases* focus on disasters to invite reflection on the systemic context of engineering, including policy effects or cultural and socio-economic models. They often call for students to take a hypothetical stance on the “kind of world they want to engineer” (Mitcham, 2017). Students study structural limitations and are encouraged to pursue responsible engineering practices by improving existing norms, policies and regulations (Swearengen & Woodhouse, 2003; Swierstra & Jelsma, 2005).

*Detached special-event micro cases* are often referred to as “disaster” cases as they present events with catastrophic consequences for individuals or the environment. There is a strong focus on accountability and prevention and the retrospective identification of the chain of causes leading up to the calamitous incident. It is one of the most popular case types in engineering-ethics instruction (Huff & Frey, 2005: 401). As the same scenarios are used for both approaches described above, in Table 1 we have pooled the examples for these two case types.

*Detached common macro cases* are concerned with the societal, cultural and political aspects of engineering (Lynch & Kline, 2000) and feature an engineering product or decision-making process, analyzing the products values and anticipated use contexts, emphasizing forward-looking reflections. Broader engineering issues such as sustainability or inequality can be explored hypothetically using this case type (Gorman et al., 2000; Kline, 2010) such that students learn how technological innovation is interwoven with a broader, complex reality.

*Detached common micro cases* are typically formulated as dilemmas individual engineers are likely to be faced with during their careers, strongly emphasizing the development of moral reasoning and knowledge of professional codes and standards. Topics tend to be derived from the precepts of professional codes of conduct, national and international regulations and health and safety standards, and may include conflicts of interest, professional integrity or safety issues (Latcha & Jordan, 1996; Shallcross, 2013).

There is a growing criticism of detached case studies (Martin et al., 2021). Due to the distant nature of engineering-ethics case instruction we struggle to sufficiently show the social dimension of engineering and the power relationships inherent to the profession (Bucciarelli, 2008; Lynch & Kline, 2000; Martin et al., 2019; Winner, 1986). So-called co-creative initiatives explore more effective approaches by using cases that reflect real-life engineering contexts and practices (Membrillo-Hernández et al., 2018; Holgaard & Kolmos, 2018; Kalamas Hedden et al., 2017; Bissett-Johnson & Radcliffe, 2021; Neto et al., 2019). Co-creation is seen as “the active involvement and engagement of actors in the production of knowledge that takes place in processes either emerging or being facilitated and designed to accomplish such active involvement” (Frantzeskaki & Kabisch, 2016, p. 91). The products, procedures or reflections that arise from the educational process are communicated widely and applied in practice (Iversen & Pedersen, 2017). Co-creative learning fosters problem ownership among students (Ryan & Tilbury, 2013), promoting shared commitment among students, tutors/coaches and external stakeholders, making the learning process a truly collaborative endeavor (Cook-Sather et al., 2014; Nieuwerburgh, 2012; Passmore, 2015; Ribes-Giner et al., 2016; van Diggelen et al., 2019). In consultation with stakeholders, students perform case-specific ethics evaluations and, if outcomes are judged ethically and technically suitable by both parties, they will co-create an-end product fit for use in the sought-after innovation process embracing decision reports, promotional/educational videos, persuasive artefacts or an improved technology.

Two case-study types can be distinguished: c*o-creative common macro cases* that promote students to take an active stance on the design of suitable strategies and engineering solutions to address broad-scale problems such as the millennium goals, and *co-creative common micro cases* where students will be collaborating with one or multiple (local) external stakeholders on the ethical and technical aspects of an authentic challenge.

### Challenge-based learning

Challenge-based learning (CBL) is one approach to the co-creative common micro case. In CBL, student learning centers on an open ended, real-life unsolved challenge for which a community of external stakeholders (companies, governments, knowledge institutions and/or citizens) seeks a solution (Kohn Rådberg et al., 2020; Malmqvist et al., 2015). Students are asked to conceive, design and implement environmental, social and/or economic solutions by using existing information or gaining new knowledge from different disciplines (Malmqvist et al., 2015; Membrillo-Hernández et al., 2019a, b). As this learning process contains a substantial degree of uncertainty, the students are expected to show or develop high levels of autonomy and self-directedness (Membrillo-Hernández et al., 2019a, b; Tang & Chow, 2020). Within this didactic context, the teacher is viewed less as an expert and more as a coach guiding students through this co-creative process (Malmqvist et al., 2015; Membrillo-Hernández et al., 2019a, b). Being a fairly recent instructional method, little evidence on CBS’s effectiveness in engineering-ethics education is available. Before analyzing the approaches used in our course, we will describe our evaluation criteria and procedure.

## Assessing the Effectiveness of Case Approaches

To determine the effectiveness of our CBL approach we will use the curriculum model of Goodlad and others (Goodlad, 1979; Bombaerts et al., 2019) describing three levels that each consist of two sub-dimensions. First, the intended curriculum level refers to the vision and underlying philosophy of a curriculum (*ideal*) and to the curriculum intentions (*formal/written*). Second, the implemented curriculum level includes the interpretation of the curriculum by the teachers (*perceived*) and the teaching as it actually happens (*operational*). Third, the attained curriculum level consists of the learning experiences by the students (*experiential*) and the resulting learning outcomes (*learned*).

Goodlad’s curriculum model indicates it might be very interesting to use the intended curriculum level and its two sub-dimensions (ideal and formal/written) to analyze the reasons to opt, implicitly or explicitly, for a certain case approach. The overview on educational objectives given in Table 1 could be an interesting starting point. Given the limitations of the article and because this is not relevant for our current analysis, we will not further analyze this. However, as mentioned in the introduction, we want to focus on staff interpretations (perceived curriculum) and operationalizations (operational curriculum) and on students’ learning experiences (experiential curriculum) and outcomes (learned curriculum).

### Perceived and Operational Curriculum

To efficiently translate the CBL principles into an actual course, teachers need to consider the instrumentality (Does it support the teaching process?), congruence (Does it fit the circumstances?) and cost (Is it feasible considering the available time and resources?) of a (re)design (Bombaerts, 2020; Doyle & Ponder, 1977; Janssen et al., 2013). A teacher’s previous experiences in teaching ethics to engineering students (e.g., frustrations or successes) and their personal views of the characteristics of the student population (e.g. approaches to learning or intellectual development) will strongly determine their course design (Felder & Brent, 2005), as will contextual factors such as the time available to develop courses, pregiven learning objectives, the type of classrooms available, student group sizes, and digital platforms (Bombaerts & Spahn, 2019). As CBL is a very open approach, its effectiveness is best evaluated using open qualitative methods such as open questions, interviews and observations.

### Experiential Curriculum: Basic Needs and Motivation

When analyzing the students’ learning experiences (experiential sub-dimension), the motivation of students to engage in the learning process is a widely used indicator. Self-determination theory (SDT), a well-established motivational model in engineering education, states that motivation is nourished by three basic needs described as “psychological nutrients that are essential for individuals’ adjustment, integrity and growth” (Ryan, 1995; Vansteenkiste et al., 2020). *Autonomy* refers to the perception of psychological freedom, choice in activities and voluntary participation. In an ethics course, students will appreciate being allowed to determine how to execute an assignment and which ethical theories to apply. *Relatedness* implies the need to feel connected to peers, tutors/coaches or external stakeholders, while *competence* denotes the feeling of being able to successfully perform an activity, have control over the outcome and experience mastery (Ryan, 1995). An ethics assignment should be designed such that students will see the task as an exciting challenge they are happy to tackle.

SDT defines motivation as a spectrum ranging from *amotivation*, with students avoiding a given task and showing disinterest in the learning experience, to *intrinsic motivation,* where students inherently value the enjoyable aspects of studying. Between these extremes, SDT distinguishes *identified regulation* where students consciously value a learning goal such that they recognize the personal importance of the task and develop a desire for self-endorsement. Even if an engineering student may not be intrinsically attracted to the ethics of their discipline, his/her aspiration to become a good engineer may prompt him/her to acknowledge that it is an essential component of the profession and to thus put in an effort to successfully complete the course.

CBL is claimed to satisfy these basic needs by fostering the students’ autonomy and self-directedness (Kohn Rådberg et al., 2020), the development of disciplinary and transversal competences (Membrillo-Hernández et al., 2019a, b) and the feeling of being part of a community that works towards a common goal (Acuńa et al., 2017). Thus, CBL can be expected to cultivate motivation for learning by rendering practical meaning to the study (Membrillo-Hernández, 2019a, b). Since high intrinsic motivation is related to beneficial behavioral outcomes such as deep learning, the aim is to optimally meet the students’ basic needs and boost motivation.

### Learned Curriculum: ACQA-Based Self-assessment of Competence Development

When analyzing the students’ learning outcomes (Goodlad’s learning sub-dimension), competences are an important indicator. We had our students assess the course using the Academic Competences and Quality Assurance (ACQA), a measure of competence development gauging competencies such as dynamic combinations of knowledge and epistemic values (Silvast et al., 2020), understanding, skills and abilities (Anderson et al., 2001). The ACQA offers a framework for the evaluation of engineering education (Meijers et al., 2005; Perrenet et al., 2017) by distinguishing seven competence domains relevant to all training programs and defining five to eight discipline-independent competencies per domain at the bachelor’s and master’s level. ACQA can be used as a teacher-rated or self-assessment tool and, being a generic measure for engineering education, can be used to compare different courses. In our evaluation we will focus on six competence domains and have reformulated the competencies to fit the engineering-ethics course evaluated (see Table 6 in the “Appendix”).

## Context: First-Year Undergraduate Course on the Ethics of Technology

We compared two approaches to a compulsory ethics-of-technology course first-year engineering students attended from April to June 2019 at Eindhoven University of Technology in the Netherlands (Bekkers & Bombaerts, 2017; Bombaerts & Doulougeri, 2019; Doulougeri & Bombaerts, 2019).

### Detached Approach

The first, detached course approach comprised theoretical lectures and a lab assignment. Students had the choice between two tracks: *Behaviour Change Technologies* and *Self-Driving Cars*. Both tracks were attended by approximately 150 students who all attended a lecture at the beginning of each week, after which they joined their tutorial group. Each group consisted of around 35 students supervised by a PhD student. The lectures covered the ethical aspects of the two topics in general terms, after which basic ethical concepts such as values and risks were introduced, leading up to major ethical theories (deontology, utilitarianism and virtue ethics) and reflections on the strengths and weaknesses of each ethical perspective.

**Fig. 1:**
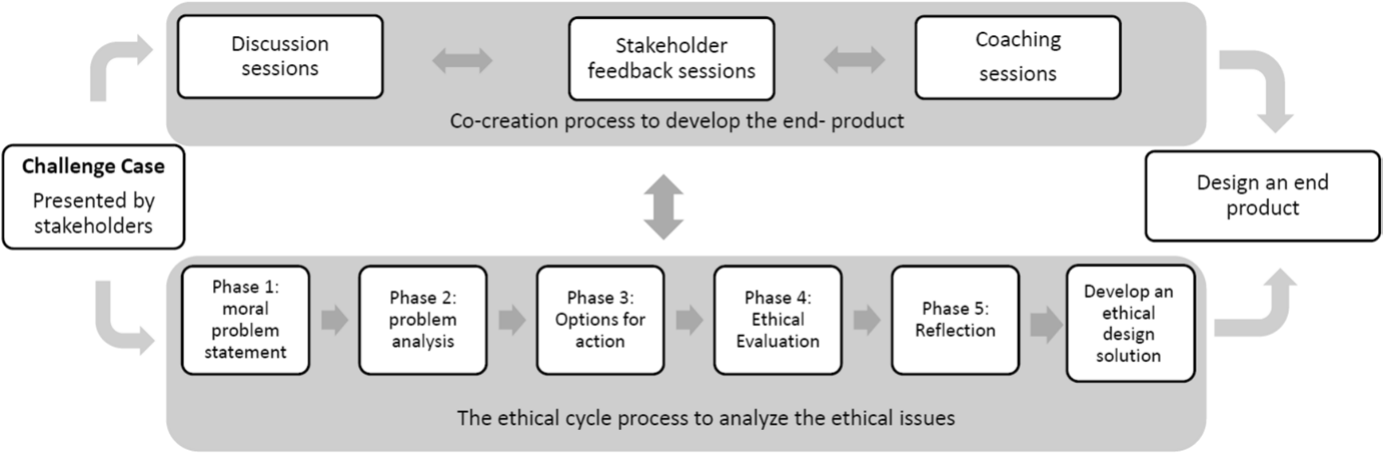
Overview of the case-based learning (CBL) process

Students worked in groups of four on a macro or micro common case study (e.g., analysizing the merits and drawbacks of health-coaching apps or programming specific crash algorithms, respectively). The groups were asked to apply the *Ethical Cycle* (van de Poel & Royakkers, 2007), a step-by-step problem-solving tool that guides students through the ethical questions of a case study (see Fig. 1 for the steps), twice during the course. In the first cycle, the students evaluated different options for actions considering ethical values and potential risks. After having received feedback on their conclusions from their peers and tutor/coach, in the second cycle they were invited to improve their first analysis based on the feedback and subsequently review the resulting report from the perspective of the three major ethical theories. Before handing in their work, they presented this draft to their peers and tutor for feedback and a final tweak.

### Challenge-Based Learning Approach

The CBL course had a total of 180 students attending in three discussion groups of 60 students, with each group comprising 12 lab groups of five students. Also implementing the Ethical Cycle, each group analyzed an ethical issue external co-creators were facing, developed a design solution that would address the problem while arguing why their solution was the most ethical. Each group was to create an end-product in any format, with the chosen format needing to show ethical sensitivity and be based on a sound analysis of their stakeholder’s ethics challenge. Each stakeholder worked with three lab groups, seeing the groups four times over the course of nine weeks. In the introductory meeting, the stakeholders gave a short presentation and during the subsequent meetings provided feedback based on the students proposals and questions. The course was concluded by an end-of-course poster presentation, with the lab groups showing their end-product to all their peers, tutor/coaches and stakeholders.

The *discussion groups* had a flipped-classroom design, with the students reading the material on ethics theories at home, while in-class time was reserved for assignments and discussions about the case and the application of the ethics models to the stakeholder’s case. During the four *stakeholder-feedback meetings* the lab groups discussed progress and asked questions. The students were expected to run the lab-group meetings autonomously, but for each meeting 15-min of *coaching time* was reserved during which their coach would provide the students with advice and feedback on the content of the assignment or their learning process (see Fig. 1).

The lab groups produced a diverse range of end-products. For example, CASA, one of the external stakeholders, presented the challenge “How can CASA use sensors in smart houses such that it respects privacy and ensures security?” Concluding that the CASA house did not pose any ethical issues if its occupants were well-informed, one group produced a promotional video that addressed autonomy and privacy in an in-depth but for laypeople understandable fashion. Another group developed Fourier transformations to change the sensor data into data that is not meaningful for future inhabitants but could still be used for acoustics analysis, thus avoiding privacy issues. The CASA team integrated both results in their further work.

## Research Questions

We expected the CBL course to foster the students’ ability to make meaningful choices (*autonomy)*, develop a sense of commitment and connection with tutors and industry partners (*relatedness*), tackle a complex task in their area of interest (*competence*), derive pleasure from the task (*intrinsic motivation*) and develop relevant engineering activities (*identified regulation*). We further anticipated a positive effect on competence development, especially with regard to the competences of problem formulation, communication, interdisciplinarity and case- and context-relevant decision-making. The first research question hence reads: “Do students in the CBL approach report higher basic needs, motivation and competence development compared to their peers in the detached approach?”.

In this exploratory inquiry, we make a first attempt at capturing the role of co-creation by analyzing the relationship between student-perceived co-creation and the other variables in the CBL group. We expected to find a strong relationship with self-reported *relatedness* and *competence development* regarding reflection, standpoint formulation, communication and interdisciplinary collaboration since these competences are thought to be specifically addressed in the CBL format. Accordingly, our second research question was: “What is the relationship between student-perceived co-creation and their self-reported basic needs, motivation and competence development?” Lastly, we sought to answer a broader, third question: “What are the implications of CBL/co-creation for ethics teaching and learning?”.

## Analysis

### Instruments

We used a mixed methods sequential explanatory design consisting of two distinct phases: a quantitative phase followed by qualitative phase to answer our queries (Creswell et al., 2003). The rationale for choosing this approach is that the quantitative data collection and analysis provided a general understanding of the research problem, while the qualitative data collection and analysis helped us refine and explain the quantitative results by exploring participants’ views in more depth (Creswell et al., 2003).

For research questions 1 and 2 we used the data collected from our custom-designed online student survey completed in weeks 1 and 9. Students rated all items on a 5-point Likert scale (ranging from 1 “Not at all” to 5 “Very much”), except for the item *overall evaluation* for which a 10-point scale was used (see Table 5 in the “Appendix”). The students judged the three items on *enjoyment*, *relevance* and *overall evaluation* at both timepoints while they rated all other items in week 9 only.

The three basic needs (*competence*, *relatedness* and *autonomy*) were assessed with a validated basic needs survey (Ilardi et al., 1993) using three items per factor. Motivation was gauged using two items per motivation type (*intrinsic motivation*, *identified regulation* and *amotivation*) taken from the validated Self-Regulation Questionnaire–Academics’ (Vansteenkiste et al., 2009). We initially developed eight items to gauge *co-creation* based upon the definition formulated by Frantzeskaki and Kabisch (2016), of which four were retained after testing their validity during informal student interviews. Taking the ACQA as a starting point, we also composed (and tested) a questionnaire to assess competence development that could serve both as an assessment tool for teacher/coaches and as an online student survey For each competence dimension, one competence was selected and modified to coincide with the ethics topic being addressed, with three items per dimension (Table 6).

For our third research interest, we collected qualitative data from the students and coaches in the CBL course. We had students answer two open questions included in the end-of-course online survey: “What did you like about the course?” and “What would you like to see changed? They moreover participated in informal 10/15-min interviews, with their experiences with the CBL and co-creation format being recorded immediately after the interview as this fosters a 'low-pressure' interaction between the researcher and student (Jorgensen, 1989). We also conducted interviews with the three coaches to learn of their experiences with the co-creation paradigm. All three had previously taught the course using a detached approach, which allowed them to compare the two methodologies.

### Procedures, Samples and Factor Analyses

All students taking the detached or CBL course received an invitation by email to fill out our electronic questionnaire, asking for informed consent and informing them they would not receive compensation for their participation. For our analyses, we received an anonymized master file, in agreement with the national law and recommendations of the university’s data protection officer.

With 10.4% of the 183 students in the CBL condition responding, the response rate was low; for the detached condition it was sufficient, with 18.0% of the 316 students returning the survey (Nulty, 2008). In week 9, 30.6% and 17.7% completed the questionnaire, respectively. Gender-distribution analysis of the two samples and the ANOVA comparing responders and non-responders across departments at both timepoints showed no significant effects, indicating the absence of gender and departmental response biases. All factors had good reliability scores (Kline, 2013): the Cronbach’s alphas for the three basic needs ranged between 0.77 and 0.91, the value for co-creation was 0.74, while competence development factors were all higher than 0.81.

In Sect. 6.3, we performed t-tests to identify differences between the two teaching approaches and computed effect sizes (Cohen’s d) to weigh the relevance of the resulting differences, with values between 0.5 > d ≥ 0.2 being classified as small, those between 0.8 > d ≥ 0.5 as medium and d > 0.8 as large (Cohen, 1988). Conclusions regarding any baseline group differences could not be drawn since university regulations did not allow us to perform any measurements prior to the courses starting. This is why we ran t-tests at the end of week 1, assuming that the students could then rely on their first impressions and experiences (enjoyment, relevance and overall impression of the course). To explore the students’ views on co-creation, we computed in Sect. 6.4 Pearson’s correlations for the data obtained in the CBL group only as the students in the detached approach had no direct experience with the method. Effects were considered small when r > 0.1, medium when r > 0.3 and large when r > 0.5 (Cohen, 1988).

In Sect. 6.5, we inspected the qualitative data pertaining to the experiential curriculum using content analysis (Jennings, 2004), taking the students’ answers to the open survey questions as our primary source of information as they contained original quotes; the notes derived from the informal interviews of 51 students served as auxiliary material (see Table 4 in the “Appendix”). The data was open-coded by reading the students’ responses several times and creating tentative labels for data sequences. Next, relationships among open codes were identified and data categorized in themes. To describe the properties of each theme, we drew on words students had used. The same procedure was applied to analyze the coaches’ responses given during the interviews, with the derived data serving as the primary source of information and the notes on observations as supportive material. Separate evaluations of the supportive material did not yield any new themes.

### Differences Between the Detached and the CBL Approach

As can be gleaned from Table 2, in week 1 we found no differences in *enjoyment* between the two approaches but in week 9 differences were significant, with a large effect size. *Relevance* showed no differences at either timepoint, while *overall evaluation* did, with an increase in means from 0.63 (*p* < 0.5, Cohen’s d = 0.54) to 0.95. Accordingly, the *relevance* factor does not inform the role of group differences prior to the course, whereas for *enjoyment* and *overall evaluation* the differences clearly increased in significance and size.

**Table 2:** The number of respondents (N), means (M), standard deviations (SD), differences in means (ΔM), significances and Cohen’s d effect sizes (d) for the factors of interest for the case-based learning (CBL) and detached course approach at end of course (week 9)

Item/Factor	CBL			Detached			Difference	
	N	M	SD	N	Mean	SD	ΔM(sign)	d
Enjoyment	57	4.02	0.79	58	2.98	0.93	1.04***	1.20
Overall evaluation	57	7.48	1.22	56	6.50	1.74	0.95**	0.63
Autonomy	55	4.27	0.63	55	3.99	0.64	0.28*	0.45
Competence	55	3.85	0.82	55	3.24	0.88	0.62***	0.73
Relatedness	55	4.01	0.63	55	4.02	0.76	− 0.01	− 0.02
Intrinsic motivation	54	3.38	0.77	54	2.76	0.97	0.62***	0.71
Identified regulation	54	2.06	0.97	54	2.93	1.07	− 0.87***	− 0.85
Amotivation	54	3.01	0.80	54	2.19	1.18	0.81***	0.81
Acqa2_reformulate	53	3.93	0.58	54	3.67	0.71	0.28**	0.44

*p < 0.05, **p < 0.01, ***p < 0.001

Of the factors assessed in week 9 only, the differences between the approaches were non-significant for *relatedness,* small for *autonomy* and medium for *competence*. The reported level of *intrinsic motivation* was higher (medium effect), that for *identified regulation* lower and for *amotivation* higher (large effect) in the CBL approach. There were no significant differences in self-perceived competence development, except for *ACQA2_reformulate* where the student/co-creators gave higher ratings, with a small effect. Thus, the two approaches had less impact on the acquisition of competences than hypothesized.

### The Role of Student-Perceived Co-creation

The correlation analyses of the qualitative data showed the degree of *perceived co-creation* to have strong positive correlations with *overall evaluation, relatedness, competence, intrinsic motivation and ACQA5_communication.* (See Table 3).

**Table 3:** Pearson’s correlations r (with significance) for basic needs, motivation, *relevance*, *overall evaluation*, and perceived competence development. Strong effect size r>0,5 in bold

Factor—r(sign)		Factor—r(sign)		Factor—r(sign)	
*Relevance*	.364^**^	***Intrinsic motivation***	.573^***^	*ACQA3_reflect*	.286*
***Overall evaluation***	.548^***^	*Identified regulation*	− .297^*^	*ACQA4_standpoint*	–
*Autonomy*	.355^**^	*Amotivation*	.475^***^	***ACQA5_communicate***	.658***
***Relatedness***	.507^***^	*ACQA1_knowledge*	.316*	*ACQA6_interdisciplinarity*	–
***Competence***	.548^***^	*ACQA2_reformulate*	.290*	*ACQA7_context*	.291*

*p < 0.05, **p < 0.01, ***p < 0.001

### Implications of CBL for Ethics Teaching and Learning

In their evaluations of the CBL approach as applied in our engineering-ethics course, the students deemed the use of the flipped-classroom design, the discussions with their coaches and stakeholders and their autonomy to be the most valuable.

Over 90% of the students interviewed reported a preference for the flipped-classroom approach, as it facilitated learning. Having to prepare the theoretical material in advance made lecture times more productive while enhancing self-regulated learning. Lecture times could now be dedicated to lab-group activities and poster presentations. As a student put it: “I enjoyed the flipped-classroom method because it permits a hands-on perspective. I still acquired the necessary knowledge, but the practical side of this course was really nice.” Since the discussion sessions adhered to the same format every week, they were judged to be somewhat repetitive towards the end of the course.

The students appreciated the time spent with their coaches as it helped them bring structure to their work. Students had anticipated they would be reporting on their progress and ask questions whereas the coaches far rather encouraged them to reflect on the overall process. The coaches were perceived as knowledgeable, warm and responsive to their needs. The coaches had supported the translation and implementation of the ethics models with the Ethical Cycle, but had also encouraged the students to look for different theories and apply them in creative ways. Rather than just remaining theories, CBL/CC had helped them turn ethics models into practical instruments to make informed design choices.

The students we interviewed were excited to work on a project with real-life stakeholders because it had enhanced their perception of the *relevance* of their challenge as their final report was deemed and treated as valuable to the real world. Most students did feel that the stakeholders were more of an add-on rather than true stakeholders since they had been attending their group only four times (introductory session, two feedback sessions and final poster presentation). The stakeholders concluded that it had been feasible to tutor three to six lab groups, even though it concerned first-year undergraduates who cannot be expected to bring in much technical know-how. They had been pleasantly surprised that students had come up with out-of-the-box solutions.

In addition, the students had experienced the course as ‘open’, which had raised their sense of *autonomy*: “It was really nice that we could come up with and develop our own project.” Towards the end of the course they did start struggling balancing the completion of the deliverable for the stakeholder and their lab reports (formal course requirement). As one student put it: “Assignments should be defined more clearly so there is not so much confusion anymore, with more details about what we are expected to do exactly.”

The coaches discerned three important differences between CBL and the detached approach. First, exemplifying the relevance of ethics was vital. Most students and external stakeholders lack the skills to reflect on real-life challenges in ethical terms. The coaches’ role in explaining how the lab assignment related to the ethics objectives of the course was critical for both students and stakeholders. The coaches and stakeholders had discussed the main issues they anticipated in advance. Although this narrowed down the students’ working scope to some extent, it did make tutoring more manageable for the coaches. Evidently, the latitude of the challenges had been sufficient since six groups completing the same assignment generated six completely different end-products. Moreover, when lab groups noted complementarity, they often started working together.

Ambiguity in CBL is crucial as a tool to challenge students. At the same time, first-year undergraduates in engineering need clear structure and adequate support. Ambiguity of the challenge and structure of the assignment do not have to contradict each other but can strengthen each other (Bombaerts et al., 2018). Methodologically, structure can still be open and abstract. The coaches provided structure by introducing the *Ethical cycle*, offering the students a step-by-step approach to solving the challenge posed. Additionally, the introductory lecture (without ethical content) already used the flipped-classroom design, giving the students the opportunity to familiarize themselves with the method. The group meetings always had the same (open) format, which predictability offered the students additional structure, while the weekly feedback meetings were key in addressing the issues the students encountered along the way. Although requiring a serious time investment, the coaches felt the additional four hours of student-contact time were well worth their effort.

Lastly, the coaches indicated that CBL requires much more work, stressing that organizing and implementing the course was an intense process, mentioning, among other aspects, that finding relevant external stakeholders and communicating and integrating the ethics challenges in the stakeholders’ queries was demanding. Universities that are considering introducing CBL need to be aware that, besides the time necessary to develop or modify course content, at the practical level the format also requires considerable investment of time and resources. Thus tutors/coaches need to allot additional time to prepare the seminar rooms for lab-group work (e.g., arranging tables to facilitate active student participation and interaction, providing equipment and material for the preparation of posters, etc.). Our coaches estimated they had invested approximatively 60% more time (prep and contact time) compared to the detached course approach, which is substantial but comparable with other practicum classes. With class time being devoted to discussing the application of theories to topics close to the PhD student-tutor’s expertise, the CBL format is particularly suitable for mentoring and tutoring by PhD students.

## Limitations

Although we used a sound evidence-informed approach with response rates and biases, validated questionnaires and strict statistical methods, we faced several challenges in measuring the impact of the two course approaches.

Firstly, our baseline group analysis lacked power as the CBL/CC sample in week 1 included too few respondents to be significant. Also, the timepoint (one week into the course) allowed us to only assess three items. Secondly, the Howthorne effect (Adair, 1984) may have played a role as both the students and coaches were aware that the pilot was more closely monitored, potentially inducing them to consciously or unconsciously modify facets of their behavior. Thirdly, the literature on challenge-based or co-creative learning is sparse, rendering it difficult to clearly delineate the various formats given that many detached learning approaches also actively involve students (“student-activating” instruction, problem-based learning). To differentiate the approaches, well-defined delineations are warranted. Lastly, since we tested the effects of co-creation-based learning for one course at our university only, our results need to be replicated in other settings and training programs.

## Conclusions

Taking these limitations into account, we feel justified in inferring several conclusions from our findings on the use of co-creative common micro cases in engineering-ethics instruction. Students’ resistance to ethics instruction is highlighted as a major challenge in engineering education (Harding et al., 2009; Romkey, 2015) as well as in the co-creative learning paradigm (Iversen & Pedersen, 2017, p. 21), with students being characterized as showing “disinterest, resistance, and difficulty learning about ethics and societal impact” (Polmear et al., 2018, p. 9). Our findings for *amotivation* and *identified regulation* were indeed the opposite to what we had expected. Nevertheless, we propose that CBL/CC is a suitable didactic method to confront engineering students with their resistance to the challenges of the “real world” and to encourage them to venture from their comfort zones. The need for clarity in instruction the students expressed coincides with the findings of Bissett-Johnson and Radcliffe (2021, p. 21), who note that “clear guidance and mentoring were required to increase the chances that learning activities would indeed help the student to find a creative answer.”

CBL and the co-creation format in particular require academic staff to “adapt their current teaching practice, and learn to adopt more relational approaches to teaching that are open, collaborative, dialogic, and democratic” (Bovill, 2020, p. 1034). The approach also involves more coaching and tutors with the right qualifications, all adding to the workload. Adequate support for educational staff hence is a prerequisite for CBL, as is employee retention. According to Bissett-Johnson and Radcliffe (2021, p. 16) by running lab-group projects more frequently, tutors become more adept at directing and coaching students, with their familiarity with themes/topics, clients and contexts increasing each year.

CBL requires teaching institutions to formulate their vision on the relevance and objectives of ethics education and convey how staff will be supported in their collaboration with external stakeholders and how the university’s ecosystem will provide for the approach (Steiner et al., 2018). The university’s recommendations for the entire academic curriculum can then inform decisions on its use in the engineering ethics program.

Despite the various empirical challenges and imperfections, the students and coaches participating in our study were enthusiastic about the co-creative design. Using a mixed methods design, we showed that, overall, CBL was more effective in meeting most of the educational goals set for the course than the detached format, with CBL fostering both the instructors’ educational and research objectives and the students’ basic needs, intrinsic motivation and communication skills*.* Our results are in line with studies examining student motivation in similar case approaches to teaching engineering ethics in higher education (Bairaktarova & Woodcock, 2017; Bucciarelli, 2008; Lynch & Kline, 2000; Martin et al., 2019; Wilson, 2013; Winner, 1986). Moreover, CBL can overcome two drawbacks associated with other case-based formats, of not providing sufficient “skill development at the two extremes, of problem finding and implementation” (Aldridge, 1994: 235) and not inducing a sense of ownership (Nakamura et al., 2011; Williams & Figueiredo, 2014). Based on the results presented, we conclude that in the context of teaching engineering ethics a CBL program in which students work as co-creators on behalf of and together with external stakeholders is promising, warranting further development and evaluation.

To help fill the gap in empirical knowledge on the topic (Yadav & Barry, 2009; Martin et al., 2021), we feel we have added to the evidence supporting the effectiveness of case-based approaches in engineering-ethics instruction. The proposed approach to evaluating the effectiveness of case studies in ethics instruction merits further investigation in the field of engineering education.

## Appendix

See Tables 4

**Table 4:** Overview of the mixed methods sequential exploratory design used

Research question	Dimension of CBL/CC curriculum assessed	Methods	Goal; contribution	Details
Do students in the CBL approach report higher basic needs, motivation and competence development compared to their peers in the detached approach?	Experiential curriculum	Student survey (self-report questionnaire): three overall questions basic needs ( items) motivation competence	To assess whether baseline cohorts are sufficiently comparable (week 1) to justify differences in week 9 To collect student ratings of basic needs satisfaction, motivation, competence development	Data collected in week 1 Detached approach: N = 19 CBL: N = 57 Data collected in week 9 Detached approach: N = 54–58 CBL: N = 53–57
What is the relationship between student-perceived co-creation and their basic needs, motivation and competence development?	Learned curriculum	Student survey: perceptions on co-creation (.. items)	To collect students’ ratings of co-creation in relation to basic needs satisfaction, motivation, competence development	Data collected in week 9 CBL: N = 53–57
What are the implications of CBL/CC for engineering ethics teaching and learning?	Experiential curriculum	Student survey: open- ended questions	To learn the students’ perceptions on CBL/CC; responses were consistent with survey results and notes from informal interviews	Data collected in week 9 N = 54
		Informal interviews with students	To collect the students’ perceptions on CBL/CC; interview notes were consistent with survey results and analysis of open questions	Data collected throughout the course N = 51
	Perceived, operational curriculum	Interviews with coaches	To collect the coaches’ perception on CBL/CC: answers were consistent with the observational notes	End-of-course interviews N = 3
All three research questions	Operational curriculum	Observation notes	To collect information about the CBL process. The information gathered by researcher echoes the experiences reported by students (experiential curriculum) and coaches (perceived curriculum)	Observations obtained during 6 lectures and 12 coaching sessions

, 5

**Table 5:** Factor items of the quantitative analysis rated on a 5-point Likert scale ranging from 1 “Not at all” to 5 “Very much”, except * rated from 1 “not at all” to 10 “Very much”

Factor or item	Items
Enjoy_W1	How do you think you will enjoy taking the USE basic course?
Relevance_W1	How do you think the USE basic course will contribute to your development as an engineer?
Overall evaluation_W1	How do you think, on a scale from 1 to 10, will you rate the USE basic course?*
Enjoy_W9	How did you enjoy taking the USE basic course?
Relevance_W9	The USE basic course contributes to my development as an engineer
Overall evaluation_W9	On a scale from 1 to 10, how would you rate the USE Basic course?*
Autonomy	I feel like I could make a lot of inputs to decide how my tasks got done I was free to express my ideas and opinions in this course I feel like I could pretty much be myself during this course
Relatedness	I really like the people I worked with in the USE course I got along with people during this course People in this course are pretty friendly towards me
Competence	Fellow students or tutors told me I am good at what I do I have been able to learn interesting new skills during this course I felt a sense of accomplishment from this course’s work
Intrinsic motivation	This course it’s fun This course is an exciting thing to do
Identified regulation	This course represents a meaningful choice to me The subjects of this course are an important life goal to me
Amotivation	I don’t see why I should study it and, frankly, I couldn’t care less I don’t know; I can’t understand why I should study it
Co-creation	We were engaged in the work of the stakeholder We contributed to the work of the stakeholder We were actively involved in the co-creation process with the stakeholder The stakeholder found our contribution useful

, 6

**Table 6:** Specification of the Academic Competences and Quality Assurance (ACQA) domains, the competence areas addressed in the two engineering ethics courses with associated course-specific items

ACQA competence area	Competencies addressed in the ethics course	Name	Items
1 Competent in one or more scientific disciplines	Understands the knowledge base of the relevant fields (theories, methods, techniques)	*ACQA1_knowledge*	I am able to describe important theoretical concepts of the relevant disciplines I am able to apply different theories of the relevant discipline about the same USE issue I am able to compare different theoretical explanations of the relevant disciplines for a USE issue
2 Competent in doing research 3 Competent in doing design	Is able to reformulate ill-structured research/design problems. Also takes account of the system boundaries in this. Is able to defend the new interpretation against involved parties	*ACQA2_reformulate*	I am able to describe the context of a USE problem, the different stakeholders involved and their interests I am able to translate an ill- structured USE problem to a clear research question I am able to justify my research question of a USE problem to the involved stakeholders
4 Scientific approach	Not addressed		
5 Basic intellectual skills	Is able (with supervision) to critically reflect on his or her own thinking, decision making, and acting and to adjust these on the basis of this reflection	*ACQA3_reflect*	I am able to describe the responsibilities of an engineer I am able to relate my own thinking, decision making, and acting to the responsibilities of an engineer I am able to adjust my own thinking, decision making, and acting in accordance with the responsibilities of an engineer
	Is able to take a standpoint with regard to a scientific argument in the field and is able to assess this critically as to its value	*ACQA4_standpoint*	I am able to find a scientific argument in a text with regard to the USE aspects of technology I am able to judge the scientific argument with regard to USE aspects of technology I am able to take a well- argued standpoint with regard to the scientific argument on USE aspects of technology
6 Competent in co-operating and communicating	Is able to communicate in writing about the results of learning, thinking and decision making with colleagues and non-colleagues	*ACQA5_communicate*	I am able to communicate in writing about USE topics with peers and tutors I am able to communicate in writing about USE topics with general audiences I am able to communicate in writing about USE topics with involved stakeholders
	Is able to work within an interdisciplinary team	*ACQA6_interdisciplinary*	I am able to contribute from my own discipline to the work of an interdisciplinary group I am able to understand the contributions from different disciplines than my own, to the work of an interdisciplinary group I am able to combine the contributions from different disciplines into the work of an interdisciplinary group
7 Takes account of temporal and social contexts	Is able to take societal aspects into account when developing technologies	*ACQA7_context*	I am able to describe the societal consequences of technological developments I am able to analyze the societal consequences of technological developments I am able to evaluate the societal consequences of technological developments

All seven ACQA competence areas are defined. It was decided to take competence areas 2 and 3 together as they are similar for the philosophy of technology course, and not to formulate items for a competence from competence area 4 as this refers specifically to the students’ own scientific discipline. Only the competences that are measured are mentioned, together with the name used to refer to these competences in this article and all the items that were used to measure the competences. More detailed information can be found in https://www.tue.nl/en/research/research-groups/philosophy-ethics/acqa/

.

## References

[CR1] Abaté CJ (2011). Should engineering ethics be taught?. Science and Engineering Ethics.

[CR2] Abraham, N. S., & Abulencia, J. P. (2011). Use of the LITEE lorn manufacturing case study in a senior chemical engineering unit operations laboratory. *Journal of STEM Education: Innovations and Research*, *12*(3), 9–16.

[CR3] Acuńa, A., Maya, M., Britton, E., & García, M. (2017). *Play Lab: Creating social value through competency and challenge based learning*. DS 88: Proceedings of the 19th International Conference on Engineering and Product Design Education (E&PDE17), Building community: Design education for a sustainable future, Oslo, Norway, 7 & 8 September 2017. https://www.designsociety.org/publication/40398/PLAY+LAB%3A+CREATING+SOCIAL+VALUE+THROUGH+COMPETENCY+AND+CHALLENGE-BASED+LEARNING

[CR4] Aldridge MD (1994). Professional practice: A topic for engineering research and instruction. Journal of Engineering Education.

[CR5] Bairaktarova D, Woodcock A (2017). Engineering student’s ethical awareness and behavior: A new motivational model. Science and Engineering Ethics.

[CR6] Barry BE, Ohland MW (2009). Applied ethics in the engineering, health, business, and law professions: A comparison. Journal of Engineering Education.

[CR7] Beever, J., & Hess, J. L. (2016). Deepwater Horizon oil spill: An ethics case study in environmental engineering. In *Paper presented at 2016* ASEE Annual *Conference & Exposition,* New Orleans, Louisiana. 10.18260/p.26647

[CR8] Bekkers R, Bombaerts G (2017). Introducing broad skills in higher engineering education: The patents and standards courses at Eindhoven University of technology. Technology & Innovation.

[CR9] Bijker WE (1997). Of bicycles, bakelites, and bulbs: Toward a theory of sociotechnical change.

[CR10] Bissett-Johnson, K., & Radcliffe, D. F. (2021). Engaging engineering students in socially responsible design using global projects. *European Journal of Engineering Education, 46*(1), 4–26.

[CR11] Bombaerts G, Spahn A (2019). Simplify! Using self-determination theory to prioritise the redesign of an ethics and history of technology course. European Journal of Engineering Education.

[CR12] Bombaerts, G. (2020). Upscaling challenge-based learning for humanities in engineering education. In *Proceedings of the 48th Annual SEFI Conference engaging engineering education*, pp. 104–114.

[CR13] Bombaerts, G., & Doulougeri, K. I. (2019). First-year engineering students’ experiences with a course of ethics and history of technology. *2019 ASEE Annual Conference & Exposition, Tampa, United States*, 18. https://pure.tue.nl/ws/portalfiles/portal/136340018/ASEE_Students_experiences_in_History_and_Ethics_of_Technology_190429_final.pdf

[CR14] Bombaerts, G., Doulougeri, K. I., & Nieveen, N. M. (2019). Quality of ethics education in engineering programs using Goodlad’s curriculum typology. In *Proceedings of the SEFI 47th Annual Conference*, pp. 1424–1436.

[CR15] Bombaerts, G., Doulougeri, K. I., Spahn, A., Nieveen, N. M., & Pepin, B. (2018). The course structure dilemma: Striving for Engineering students’ motivation and deep learning in an ethics and history course. In *Proceedings of the 46th SEFI Annual Conference 2018*, pp. 79–87.

[CR16] Bovill C (2020). Co-creation in learning and teaching: The case for a whole-class approach in higher education. Higher Education.

[CR17] Bucciarelli LL (2008). Ethics and engineering education. European Journal of Engineering Education.

[CR18] Byrne EP (2012). Teaching engineering ethics with sustainability as context. International Journal of Sustainability in Higher Education.

[CR19] Cohen J (1988). Statistical power analysis for the behavioral sciences. L.

[CR20] Colby A, Sullivan WM (2008). Ethics teaching in undergraduate engineering education. Journal of Engineering Education.

[CR21] Cook-Sather A, Bovill C, Felten P (2014). Engaging students as partners in learning and teaching: A guide for faculty.

[CR22] Creswell, J., Clark, V., Gutmann, M., & Hanson, W. (2003). Advance mixed methods research designs. In *Handbook of mixed methods in social and behavioral research* (pp. 209–240).

[CR23] Davis M (1999). Ethics and the university.

[CR24] Dempsey J, Stamets J, Eggleson K (2017). Stakeholder views of nanosilver linings: Macroethics education and automated text analysis through participatory governance role play in a workshop format. Science and Engineering Ethics.

[CR25] van Diggelen MR, Doulougeri KI, Gomez-Puente SM, Bombaerts G, Dirkx KJH, Kamp RJA (2019). Coaching in design-based learning: A grounded theory approach to create a theoretical model and practical propositions. International Journal of Technology and Design Education.

[CR26] Doulougeri, K., & Bombaerts, G. (2019). The influence of learning context on engineering students’ perceived basic needs and motivation. In *Paper presented at 2019 ASEE Annual Conference & Exposition, Tampa, United States*.

[CR28] Doyle W, Ponder GA (1977). The practicality ethic in teacher decision-making. Interchange.

[CR29] Felder RM, Brent R (2005). Understanding student differences. Journal of Engineering Education.

[CR31] Fotheringham, H. (2008). Ethics case studies: Placing ethical practice in an engineering context. *Innovation, good practice and research in engineering education–The Higher Education Academy Engineering Subject Centre and the UK Centre for Materials Education EE2008, Liverpool*.

[CR32] Frantzeskaki N, Kabisch N (2016). Designing a knowledge co-production operating space for urban environmental governance—Lessons from Rotterdam, Netherlands and Berlin, Germany. Environmental Science & Policy.

[CR33] Gorman, M. E., Mehalik, M. M., & Werhane, P. H. (2000). *Ethical and environmental challenges to engineering*. Saddle River, N.J.: Prentice Hall.

[CR34] Guntzburger Y, Pauchant TC (2014). Complexity and ethical crisis management: A systemic analysis of the Fukushima Daiichi nuclear disaster. Journal of Organizational Effectiveness: People and Performance.

[CR35] Harding, T., Sutkus, J., Finelli, C., & Carpenter, D. (2009). Engineering culture and the ethical development of undergraduate students. In *Proceedings of the Research in Engineering Education Symposium 2009*. QLD: Palm Cove.

[CR36] Haws DR (2001). Ethics instruction in engineering education: A (mini) meta-analysis. Journal of Engineering Education.

[CR37] Herkert JR (2000). Engineering ethics education in the USA: Content, pedagogy and curriculum. European Journal of Engineering Education.

[CR99] Herkert, J. R. (2005). Ways of thinking about and teaching ethical problem solving: Microethics and macroethics in engineering. *Science and Engineering Ethics, 11*(3), 373–385. 10.1007/s11948-005-0006-3 10.1007/s11948-005-0006-3 16190278

[CR38] Herreid CF (1994). Case studies in science–a novel method of science education. Journal of College Science Teaching.

[CR39] Herreid CF (2007). Start with a story: The case study method of teaching college science.

[CR40] Holgaard, J. E., & Kolmos, A. (2018). Differences in company projects-a way of inspiring educational design for emplyability. In R. Clark, P. Munkebo Hussmann, H-M. Järvinen, M. Murphy, & M. Etchells Vigild (Eds.), *Proceedings of the 46th SEFI Annual Conference 2018: Creativity, innovation and entrepreneurship for engineering education excellence* (pp. 216–223). Brussels: European Society for Engineering Education.

[CR41] Huff C, Frey W (2005). Moral pedagogy and practical ethics. Science and Engineering Ethics.

[CR42] Ilardi B, Leone D, Kasser T, Ryan R (1993). Employee and supervisor ratings of motivation—main effects and discrepancies associated with job-satisfaction and adjustment in a factory setting. Journal of Applied Social Psychology.

[CR43] Iversen, A-M., & Pedersen, A. S. (2017). Co-creating knowledge: Students and teachers together in a field of emergence. In T. Chemi, & L. Krogh (Eds.), *Co-creation in higher education: Students and educators preparing creatively and collaboratively to the challenge of the future*. Brill | Sense. Creative Education Bookseries, 6, 15–30.

[CR44] Janssen F, Westbroek HB, Doyle W, Van Driel JH (2013). How to make innovation practical. Teachers College Record.

[CR45] Jennings GR (2004). Business and social science methods.

[CR46] Jonassen DH, Hernandez-Serrano J (2002). Case-based reasoning and instructional design: Using stories to support problem solving. Educational Technology Research and Development.

[CR47] Jorgensen DL (1989). Participant observation: A methodology for human studies.

[CR48] Kalamas Hedden M, Worthy R, Akins E, Slinger-Friedman V, Paul RC (2017). Teaching sustainability using an active learning constructivist approach: Discipline-specific case studies in higher education. Sustainability.

[CR49] Kauffmann, P., Abdel-Salam, T., Williamson, K., & Considine, C. (2005). Privatization initiatives: A source for engineering economy case studies. *Paper presented at the 2005 American Society for Engineering Education Annual Conference & Exposition,* Salt Lake City, Utah.

[CR50] Kline RR (2010). Engineering case studies: Bridging micro and macro ethics. IEEE Technology and Society Magazine.

[CR51] Kline P (2013). Handbook of psychological testing.

[CR52] Kohn Rådberg K, Lundqvist U, Malmqvist J, Hagvall Svensson O (2020). From CDIO to challenge-based learning experiences—expanding student learning as well as societal impact?. European Journal of Engineering Education.

[CR53] Kumar D (2015). Consumer behaviour: Includes online buying trends.

[CR54] Latcha, M., & Jordan, W. (1996). To ship or not to ship: An engineering ethics case study. In *Technology-based re-engineering engineering education proceedings of frontiers in education FIE’96 26th Annual Conference*, *3*, 1159–1163.

[CR55] Lundeberg, M. A. (2008). *Case pedagogy in undergraduate STEM: Research we have; research we need*. Paper commissioned by the Board on Science Education, National Academy of Sciences.

[CR56] Lynch WT, Kline R (2000). Engineering practice and engineering ethics. Science, Technology, & Human Values.

[CR57] Malmqvist, J., Rådberg, K. K., & Lundqvist, U. (2015). Comparative analysis of challenge-based learning experiences. In *Proceedings of the 11th International CDIO Conference, Chengdu, China*. https://research.chalmers.se/en/publication/218615

[CR58] Martin DA, Conlon E, Bowe B (2019). The role of role-play in student awareness of the social dimension of the engineering profession. European Journal of Engineering Education.

[CR100] Martin, D. A., Conlon, E. & Bowe, B. (2021). Using case studies in engineering ethics education: The case for immersive scenarios through stakeholder engagement and real life data. *Australasian Journal of Engineering Education.*10.1080/22054952.2021.1914297.

[CR59] McCarton, L., & O'Hógáin, S. (2018). Where there is no engineer - Designing for community resilience. Development Technology in the Community (DTC) Research Group, Technological University Dublin (DIT) & Engineers without Borders (EWB) Ireland. Retrieved from https://arrow.tudublin.ie/cgi/viewcontent.cgi?article=1006&context=engschivbk.

[CR60] Meijers, A. W. M., van Overveld, C. W. A. M., & Perrenet, J. C. (2005). *Criteria for academic bachelor’s and master’s curricula*. https://www.utwente.nl/en/ces/celt/toolboxes/educational-design/1a_course_embedded_in_curriculum/criteria-for-academic-bachelors-and-masters-curricula.pdf

[CR61] Membrillo-Hernández J, Muñoz-Soto RB, Rodríguez-Sánchez ÁC, Díaz-Quiñonez JA, Villegas PV, Castillo-Reyna J, Ramírez-Medrano A (2019). Student engagement outside the classroom: analysis of a challenge-based learning strategy in biotechnology engineering. IEEE Global Engineering Education Conference (EDUCON).

[CR62] Membrillo-Hernández J, Ramírez-Cadena JM, Martínez-Acosta M, Cruz-Gómez E, Muñoz-Díaz E, Elizalde H (2019). Challenge based learning: The importance of world-leading companies as training partners. International Journal on Interactive Design and Manufacturing (IJIDeM).

[CR63] Mitcham, C. (2017). Engineering ethics: From thinking small to deep and big. *Colorado School of Mines 2017 Faculty Senate Distinguished Lecture*. Retrieved from https://www.mines.edu/faculty-senate/lecture/2017-mitcham/.

[CR64] Neto OM, Lima R, Mesquita D (2019). Changing an engineering curriculum through a co-construction process: A case study. The International Journal of Engineering Education.

[CR65] Newberry B (2010). Katrina: Macro-ethical issues for engineers. Science and Engineering Ethics.

[CR66] Passmore J (2015). Excellence in coaching: The industry guide.

[CR67] Perrenet J, Borghuis T, Meijers A, van Overveld K, Mulder M (2017). Competencies in higher education: Experience with the academic competences and quality assurance (ACQA) framework. Competence-based vocational and professional education: Bridging the worlds of work and education.

[CR68] Pesch U, Huijts NMA, Bombaerts G, Doorn N, Hunka A (2020). Creating ‘local publics’: Responsibility and involvement in decision-making on technologies with local impacts. Science and Engineering Ethics.

[CR69] van de Poel I, Royakkers L (2007). The ethical cycle. Journal of Business Ethics.

[CR70] Polmear, M., Bielefeldt, A. R., Knight, D., Swan, C., & Canney, N. (2018). Faculty perceptions of challenges to educating engineering and computing students about ethics and societal impacts. *American Society for Engineering Education Annual Conference & Exposition*, 18.

[CR71] Ribes-Giner G, Perelló Marín MR, Pantoja-Diaz O (2016). Co-creation impacts on student behavior. Procedia-Social and Behavioral Sciences.

[CR72] Romkey, L. (2015). Engineering, society and the environment in the teaching goals and practices of engineering instructors. In *Proceedings of the American Society for Engineering Education (ASEE) Annual Conference and Exposition.* Seattle WA.

[CR73] Ryan RM (1995). Psychological needs and the facilitation of integrative processes. Journal of Personality.

[CR74] Ryan A, Tilbury D (2013). Flexible pedagogies: New pedagogical ideas.

[CR75] Shallcross DC (2013). Safety education through case study presentations. Education for Chemical Engineers.

[CR76] Silvast A, Laes EJW, Abram S, Bombaerts G (2020). What do energy modellers know?: An ethnography of epistemic values and knowledge models. Energy Research and Social Science.

[CR77] Steiner SD, Brock DD, Pittz TG, Liguori E (2018). Multi-disciplinary involvement in social entrepreneurship education: A uniquely threaded ecosystem. Journal of Ethics & Entrepreneurship.

[CR78] Swearengen JC, Woodhouse EJ (2003). Overconsumption as an ethical challenge for engineering education. International Journal of Mechanical Engineering Education.

[CR79] Swierstra, T., & Jelsma, J. (2005). Trapped in the duality of structure: An STS approach to engineering ethics. In H. Harbers, & J. A. Harbers (Eds.), *Inside the politics of technology. Agency and normativity in the co-production of technology and society* (pp 199–227). Amsterdam University Press.

[CR80] Tai D-Y (2013). Engineering ethics, STS, and the China airlines CI-611 accident. East Asian Science, Technology and Society: An International Journal.

[CR81] Tang ACY, Chow MCM (2020). To evaluate the effect of challenge-based learning on the approaches to learning of Chinese nursing students: A quasi-experimental study. Nurse Education Today.

[CR82] Thiel CE, Connelly S, Harkrider L, Devenport LD, Bagdasarov Z, Johnson JF, Mumford MD (2013). Case-based knowledge and ethics education: Improving learning and transfer through emotionally rich cases. Science and Engineering Ethics.

[CR83] Vansteenkiste M, Ryan RM, Soenens B (2020). Basic psychological need theory: Advancements, critical themes, and future directions.

[CR84] Vansteenkiste M, Sierens E, Soenens B, Luyckx K, Lens W (2009). Motivational profiles from a self-determination perspective: The quality of motivation matters. Journal of Educational Psychology.

[CR85] Vaughan D (1996). The Challenger launch decision: Risky technology, culture, and deviance at NASA.

[CR86] Velasquez, M. G. (1992). The Ford motor car. In *Business ethics: Concepts and cases*, 3rd ed (pp. 110–113). Englewood Cliffs.

[CR87] Wilson WR (2013). Using the Chernobyl incident to teach engineering ethics. Science and Engineering Ethics.

[CR88] Winner L (1986). Do artefacts have politics? ’First published in Daedalus, reprinted in The Whale and The Reactor.

[CR89] Yadav A, Barry BE (2009). Using case-based instruction to increase ethical understanding in engineering: What do we know? What do we need?. International Journal of Engineering Education.

[CR90] van Nieuwerburgh, C. (2012). *Coaching in education: Getting better results for students, educators, and parents*. Karnac Books.

[CR91] van de Poel, I., & Royakkers, L. (2011). *Ethics, technology, and engineering: An introduction*. Wiley.

